# Intriguing Balancing Selection on the Intron 5 Region of *LMBR1* in Human Population

**DOI:** 10.1371/journal.pone.0002948

**Published:** 2008-08-13

**Authors:** Fang He, Dong-Dong Wu, Qing-Peng Kong, Ya-Ping Zhang

**Affiliations:** 1 State Key Laboratory of Genetic Resources and Evolution, Kunming Institute of Zoology, Chinese Academy of Sciences, Kunming, China; 2 Laboratory for Conservation and Utilization of Bio-resource, Yunnan University, Kunming, China; 3 Graduate School of the Chinese Academy of Sciences, Beijing, China; University of Glasgow, United Kingdom

## Abstract

**Background:**

The intron 5 of gene *LMBR1* is the cis-acting regulatory module for the sonic hedgehog (*SHH*) gene. Mutation in this non-coding region is associated with preaxial polydactyly, and may play crucial roles in the evolution of limb and skeletal system.

**Methodology/Principal Findings:**

We sequenced a region of the *LMBR1* gene intron 5 in East Asian human population, and found a significant deviation of Tajima's D statistics from neutrality taking human population growth into account. Data from HapMap also demonstrated extended linkage disequilibrium in the region in East Asian and European population, and significantly low degree of genetic differentiation among human populations.

**Conclusion/Significance:**

We proposed that the intron 5 of *LMBR1* was presumably subject to balancing selection during the evolution of modern human.

## Introduction

After dispersal from African, modern human migrated to the rest of the world and adapted rapidly to a variety of environmental challenges, such as climate, food supply, etc. Many phenotypes may undergo apparent adaptation [1]. Skeletal system has been observed to evolve rapidly during the past 10,000 years among human populations during the time of rapid population growth [2]. However, unlike other beneficial traits, e.g. speech, cognitive ability, diverse skin and others [1], virtually no single gene has been identified under a major selection event accounting for the rapid evolution of skeletal system of human.

Polydactyly, characterized by the addition or part of a digit, is one of the most common congenital limb malformations which are relatively common human abnormalities occurring at an incidence of one per ∼500–1000 live births [3]. Polydactyly occurs in many phenotypes, like preaxial polydactyly II (PPD II), triphalangeal thumb-polysyndactyly syndrome (TPT-PS) and isolated triphalangeal thumb (OMIM 174500) [3]–[7]. *LMBR1* gene, which contains 17 exons spanning approximately 200 kb of genomic DNA, encodes a 490-amino acid protein containing 9 putative transmembrane and one coiled-coil domains [8]. Level of *LMBR1* activity had been associated with the number of digits across vertebrates [4]. The crucial functional element of *LMBR1* is located within the intron 5, which serves as the long-range regulatory element of the adjacent *SHH* gene, a key development gene in the nervous system, skeletal system and others. Disruption of this intron, leading to dysregulation of *SHH*, can cause all kinds of above mentioned polydactyly [3]–[7]. Phylogenetic analysis also indicated conservation of the intron 5 region in teleost fishes and throughout the tetrapod lineage [9].

Considering the profound role of *LMBR1* gene, particularly the intron 5, in the development of limb and skeletal system, we checked the evolutionary pattern by sequencing a ∼10 kbp region in the intron 5 in 41 East Asian individuals. Tajima's D value is significantly higher than neutrality as after considering human population growth. Additionally, the advent of large-scale genome and polymorphism data in human population supports specific selection effect during human evolution. Extensive linkage disequilibrium and lower genetic differentiation were found in this region in East Asian and European populations. We concluded that balancing selection at the region occurred during the evolution of modern human.

## Results

### Genetic variation of the sequenced region in the intron 5 in the East Asians

**Figure 1:**
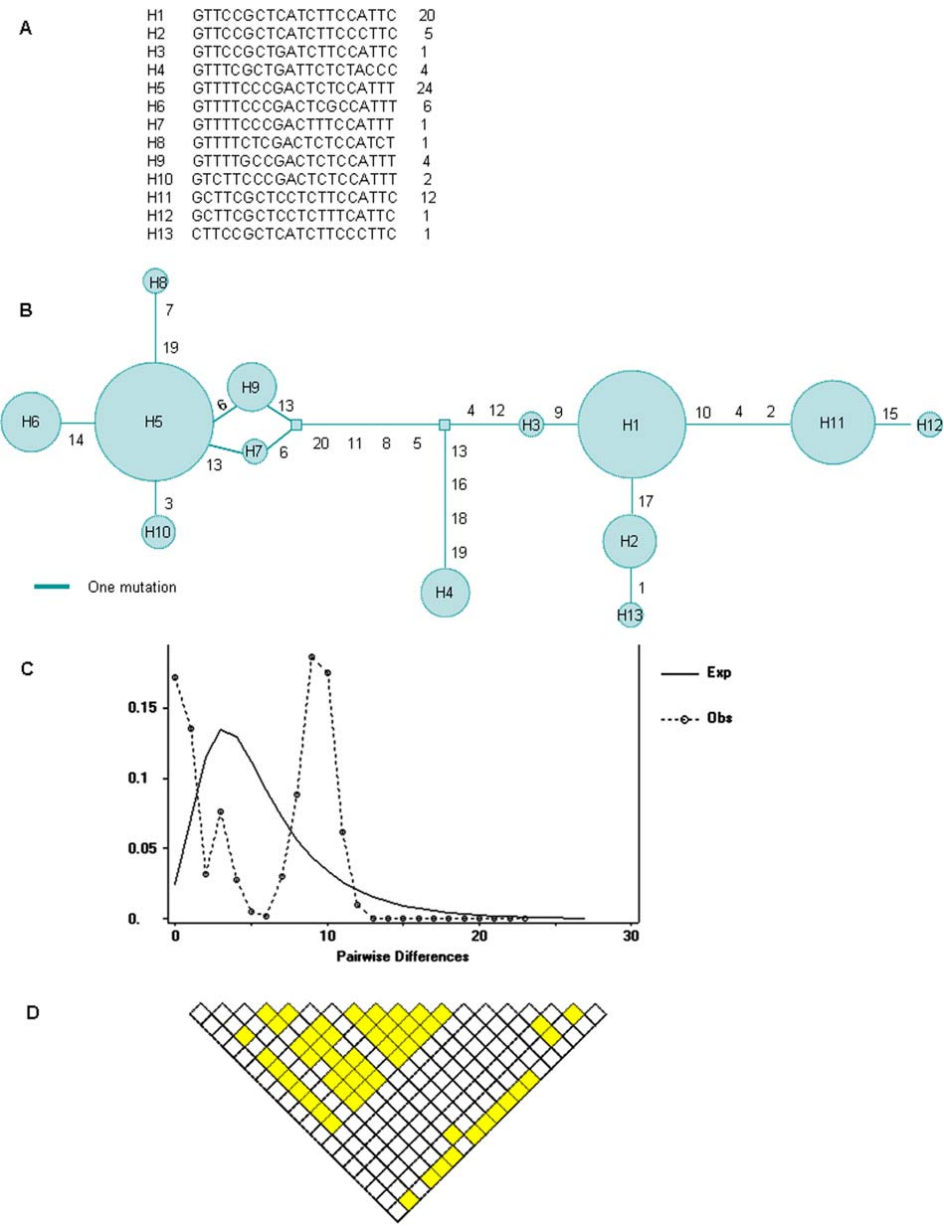
Genetic variation analyses in the *LMBR1* intron 5 among 41 East Asian individuals. A: The 13 haplotypes constructed by PHASE program, and the right-most column shows the number of each haplotypes among 41 subjects. B: Median joining network of haplotypes. Each circle represents a haplotype indicated in Figure 1A, and the size of the circle is the relative frequency. Beside the branches are labels of the SNPs in the haplotypes counted from left to right. C: Graph of pairwise differences between the haplotypes. The dash line represent the observed sequence pairwise difference, and the real line represent the expected distribution of pairwise difference simulated by DnaSP under population growth with initial theta as 3.442, final theta as 1000, and final tau as 2.267. The “twin-peak” of observed mismatch distribution is suggestive of balancing selection. D: LD extent analyzed by R^2^ of all pairwise comparisons between the 20 SNPs. The shadows indicate significant pairwise comparison identified with χ^2^ tests by using a Bonferroni correction for multiple testing.

We sequenced one 9256 bp region in the 5th intron of *LMBR1* gene in 41 East Asian individuals, and identified 21 SNPs, 8 of which had the minor allele frequencies >0.4. The sequence for each individual was submitted to GenBank under accession numbers EU880543-EU880583. One SNP was difficult to be confirmed in about one fourth individuals for sequencing technology difficulties, the SNP and 300 bp sequence around it were excluded from analysis. The other total 20 SNPs were used to construct the haplotypes by the PHASE program [10], [11], and 13 haplotypes were obtained (Figure 1A). All SNPs were not deviated from Hardy-Weinberg equilibrium.

The nucleotide diversity π is 6.4×10^−4^, lower than the human genome average (∼7.5×10^−4^) [12], and the Watterson's estimator θw is 4.49×10^−4^. The haplotype diversity is 0.828. There are two major haplotypes found at high frequencies, and the network analysis indicates that they are separated by relative long branch length (Figure 1B). The “twin-peak” phenomenon is observed in the mismatch distribution of pairwise differences between the haplotypes (Figure 1C). Tajima's D value is 1.25 (p>0.1), but the value is significantly higher at the empirical 95% level (by one sided p-value) in an empirical distribution available from a study of 313 genes [13]. These data are suggestive of the direction of balancing selection.

Evidences have suggested that human populations have grown dramatically, which have strong effects on the genetic diversity. The assumption of constant population size is highly conservative in the detection of balancing selection, and will cause type II statistical error (i.e. a failure to reject the null hypothesis of neutrality when it is false) [14]. Considering the population size change in human populations, we performed 250 different tests of Tajima's D using the algorithm of Rogers [15] across different magnitudes of population growth (from 1-fold growth [i.e., no growth] to 250-fold growth), with the growth beginning at different times (0 years ago to 250,000 years ago). The hypothesis of neutrality is rejected by Tajima's D under those models assuming magnitudes of growth greater than 1.3, from an ancient effective population size of 10,000, beginning more than about 65,000 years ago. The sequence is large enough that recombination may have occurred, and it would make Tajima's D test conservative.

### Age estimation

The time of the most recent common ancestor (T_MRCA_) is calculated as T_HC_*D_H_/D_HC_. T_HC_ is the time of divergence between human and chimpanzee, D_HC_ is the divergence between human and chimpanzee sequences (from NCBI) and D_H_ is the average difference of human haplotypes. The values are 6×10^6^ years, 95.00, and 5.71 respectively, and the T_MRCA_ is 3.61×10^5^ years, much longer than 65,000 years.

### Extensive linkage disequilibrium of the intron 5 in the East Asian and European populations

**Figure 2:**
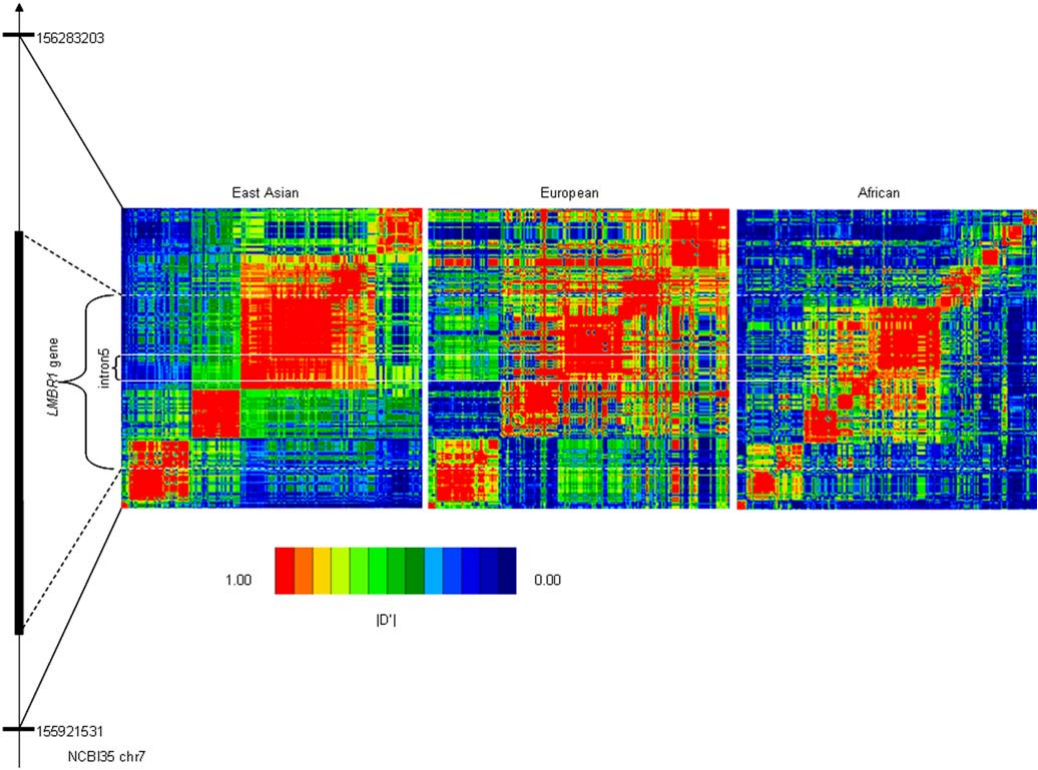
Linkage disequilibrium pattern of chr7: 155920–156290 kbp (NCBI35) region in the East Asian, European and African populations based on the HapMap Data. *LMBR1* gene and the intron 5 are showed.

Extended linkage disequilibrium is usually observed in the selected region because recombination does not have enough time to break it down [16]. Large scale polymorphism data are facilitating the studies of evolutionary patterns in human genome. We examined the characteristics of *LMBR1* by the HapMap data. All pairwise D' measures among these HapMap SNPs were estimated, and the graphical representation of LD level is illustrated in Figure 2, which demonstrates strong LD of the intron 5 region in the East Asian and European populations (Figure 2). We also estimate the LD extent by analyzing the R^2^ of all pairwise comparisons between the 20 SNPs, and found 56 significant pairs at 5% level (Figure 1D).

### Lower genetic differentiation among human populations

In general, positive directional selection promotes the divergence among populations, but balancing selection will decrease the differentiation of selected loci compared with neutrality. The average Fst values of total 113 SNPs in the *LMBR1* gene were 0.036 (East Asian vs European), 0.081 (East Asian vs African), and 0.064 (European vs African), significantly lower than the average values in [17], 0.098, 0.128 and 0.102 respectively by t test (p = 9.36E-44, 1.57E-07, 2.08E-06 respectively with degree of freedom 112; p = 0.00098, 0.016, p = 0.02 respectively with degree of freedom 2; and p = 0.014, 0.058, 0.065 respectively with degree of freedom 1). Such weak genetic differentiation indicates balancing selection might have occurred in this region rather than population subdivision.

## Discussion

**Figure 3:**
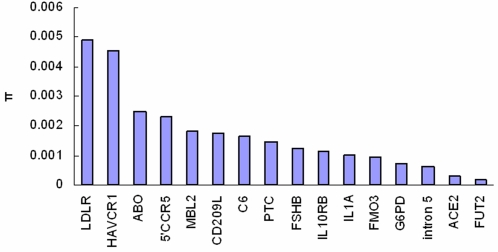
Nucleotide diversity (π) of previous reported balancing selection genes and the *LMBR1* intron 5 studied here. It shows that *LMBR1* intron 5 had low π among these genes with documented evidence of balancing selection. The data are from [29] (*LDLR*), [30] (*HAVCR1*), [18] (*ABO*, *IL10R*B, *IL1A*, and *ACE2*), [19] (5′ *CCR5*), [31] (*MBL2*), [32] (*CD209L*), [33] (*C6*), [13] (*PTC*), [34] (*FSHB*), [35] (*FMO3*), [36] (*G6PD*), [37] (*FUT2*).

Evidences have indicated that the size of human population increased in the Upper Pleistocene [15]. Populations that have grown are expected to have an excess of low-frequency alleles and thus low pairwise difference between sequences, which will lead to the reduction of common statistics used to detect from neutrality, e.g. Tajima's D, [18]. Therefore, it is inappropriate to detect natural selection, e.g. conservative to detect balancing selection, under the model of constant population size [14]. In this study, we identified significant deviation of Tajima's D from neutrality under models incorporating different human population growth parameters. Another two pieces of evidences, strong linkage disequilibrium and lower genetic differentiation among human ethnical populations also support the existence of a balancing selection, because population subdivision, another competing hypothesis, could also lead to significantly high Tajima's D for divergent haplotypes existing in different geographical regions [14], [19]. However, the nucleotide diversity is low in the region, which is not usually observed in the genes under balancing selection (Figure 3). Perhaps, the intron 5 region is highly conservative during evolution for its essential function and does not allow accumulation of new mutations. For example, the nucleotide diversity of *ACE2* gene, subject to balancing selection, is even lower than that found in this intron [20].

Cis-regulating modules have been known contributing profoundly to the genome evolution as they are key regulator of gene expression level. Up to now, cis-regulatory regions of many genes showed evidence of positive and/or balancing selection. For example, the cis-regulator of *CCR5* [21] was involved in a strong balancing selection, whereas, the encoding sequence of the gene was subject to positive selection. The intron 5 and the downstream *SHH* gene appear evolving in a similar manner as *CCR5*, in that evidence of positive selection has been found in *SHH* gene in primate, particularly lineage leading to human, and human population [22].

It is appealing that testing for selection should be based on a strong biological working hypothesis [23]. The lower genetic differentiation of the loci among human populations indicated that the balancing selection had occurred before the divergence between the main modern human subpopulations. Accordingly, we proposed that polydactyly has maintained for a long time during the evolution of human. However, considering the strangeness appearance of the phenotype, it may be a slightly side effect and disadvantage during the adaptive evolution of limb and skeletal system, like limb size, hand bone strength, finger flexibility or others. Presumably, the variants maintained by balancing selection may be associated additional intermediate phenotypes other than polydactyly. In addition, the balancing selection on the region probably also plays a role in maintaining the diversity of skeletal system, e.g. sizes, among different populations and different individuals. Although we showed the evidence of balancing selection in this region, the mechanism under which balancing selection occurred at the intron 5 of *LMBR1* gene is unclear. It may become more apparent when more insight into function of the intron 5 is available by the future functional studies.

## Materials and Methods

### Samples and sequencing

41 unrelated East Asian individuals (19 Han Chinese, 15 minorities of China, five Thais, 1 Filipino and 1 Lao) were chosen for sequencing. Ethical approval for this study was provided by the Ethics Committee of Kunming Institute of Zoology, Chinese Academy of Sciences, and all participants provided written informed consent. The first ten kbps of *LMBR1* intron 5 was amplified by LA-PCR method with two pair primers (5′-AGAAAGGAGGTCATTGTAG-3′ as first sense primer and 5′-AGATTGAGGTCCAGGTAT-3′ as first antisense primer; 5′-CGTATGGGAACTCAGAAA-3′ as second sense primer and 5′-ACGCAAGCCAAATAAGAC-3′ as second antisense primer), and sequenced by ABI PRISM 3730xl DNA analyser (Applied Biosystems) with ABI BigDye Terminator Cycle Sequencing Kit, Version 3.1 (Applied Biosystems, Foster City, California, USA). The thermal cycling condition of two pair primers both are: 95°C, 4 min; 94°C, 1 min, 57°C, 5 min, 72°C, 5 min, 30 cycles; 72°C, 10 min. The resulting sequences were analyzed by the DNASTAR software (DNASTAR). Detailed information on sequencing primer sequences is available on request.

### Statistical analysis on the sequenced intron 5 region

The haplotypes were constructed by PHASE program [10], [11], and the network was constructed by using median joining algorithm [24] implemented in Network. The average number of pairwise difference (π), Watterson's estimator (θw) [25] and haplotype diversity were calculated. Tajima's D (1989) [26] was used to test the evolutionary pattern by Arlequin program [27]. Allowing for human population growth, we applied the Tajima's D test on the background of different magnitudes of population growth and the growth beginning at different times simulated by the algorithm of Rogers (1995) [15]. Pairwise mismatch analysis was performed by DnaSP [28] under population growth assumptions with initial theta as 3.442, final theta as 1000, and final tau as 2.267. LD extent was analyzed by R^2^ of all pairwise comparisons between SNPs, and the significances were identified with χ^2^ tests by using a Bonferroni correction for multiple testing. The nucleotide diversity (π) values of previous reported balancing selection genes were obtained from [29] (*LDLR*), [30] (*HAVCR1*), [20] (*ABO*, *IL10R*B, *IL1A*, and *ACE2*), [21] (5′ *CCR5*), [31] (*MBL2*), [32] (*CD209L*), [33] (*C6*), [14] (*PTC*), [34] (*FSHB*), [35] (*FMO3*), [36] (*G6PD*), [37] (*FUT2*).

### LD analysis and Fst comparison based on the HapMap data

SNPs of *LMBR1* location in chr7:155920–156290 kbp (NCBI35) were chosen from HapMap with the criteria: minor allele frequency ≥10% and consistent with Hardy-Weinberg equilibrium in 0.01 level. LD measures between pairs of SNPs were quantified using statistic D' [37], which were calculated by Haploview program [39]. The results of pairwise D' were visualized by the GOLD program [40]. SNPs in the *LMBR1* gene region were used to calculate Fst values between Caucasians, Africans, and East Asians. Total 113 SNPs in the *LMBR1* region fulfilled the criteria were used to calculate the Fst values among human populations as described in [17], [41].
